# Overdose Detection Among High-Risk Opioid Users Via a Wearable Chest Sensor in a Supervised Injecting Facility: Protocol for an Observational Study

**DOI:** 10.2196/57367

**Published:** 2024-09-10

**Authors:** Basak Tas, Will Lawn, Marianne Jauncey, Mark Bartlett, Paul Dietze, Daniel O'Keefe, Nico Clark, Bruce Henderson, Catriona Cowan, Osian Meredith, John Strang

**Affiliations:** 1 National Addiction Centre Institute of Psychiatry, Psychology and Neuroscience King's College London London United Kingdom; 2 Department of Psychology Institute of Psychiatry, Psychology & Neuroscience King's College London London United Kingdom; 3 Medically Supervised Injecting Centre Sydney Australia; 4 Disease Elimination Burnet Institute Melbourne Australia; 5 Royal Melbourne Hospital Melbourne Australia; 6 PneumoWave LTD Motherwell United Kingdom; 7 South London and Maudsley NHS Foundation Trust London United Kingdom

**Keywords:** wearable sensor, overdose, opioid-related deaths, injecting opioid use, medically supervised injection center, opioid induced respiratory depression, mobile phone, opioid overdose, drug overdose, Australia, United States, chest biosensor, biosensor, wearable device, respiratory depression, algorithm, detection algorithm, observational design, illicit drugs, safe injecting facilities, naloxone, wearable

## Abstract

**Background:**

Opioid overdose is a global health crisis, affecting over 27 million individuals worldwide, with more than 100,000 drug overdose deaths in the United States in 2022-2023. This protocol outlines the development of the PneumoWave chest biosensor, a wearable device being designed to detect respiratory depression in real time through chest motion measurement, intending to enhance early intervention and thereby reduce fatalities.

**Objective:**

The study aims to (1) differentiate opioid-induced respiratory depression (OIRD) from nonfatal opioid use patterns to develop and refine an overdose detection algorithm and (2) examine participants’ acceptability of the chest biosensor.

**Methods:**

The study adopts an observational design over a 6-month period. The biosensor, a small device, will be worn by consenting participants during injecting events to capture chest motion data. Safe injecting facilities (SIF) in Melbourne, Victoria (site 1), and Sydney, New South Wales (site 2), which are legally sanctioned spaces where individuals can use preobtained illicit drugs under medical supervision. Each site is anticipated to recruit up to 100 participants who inject opioids and attend the SIF. Participants will wear the biosensor during supervised injecting events at both sites. The biosensor will attempt to capture data on an anticipated 40 adverse drug events. The biosensor’s ability to detect OIRD will be compared to the staff-identified events that use standard protocols for managing overdoses. Measurements will include (1) chest wall movement measured by the biosensor, securely streamed to a cloud, and analyzed to refine an overdose detection algorithm and (2) acute events or potential overdose identified by site staff. Acceptability will be measured by a feedback questionnaire as many times as the participant is willing to throughout the study.

**Results:**

As of April 2024, a total of 47 participants have been enrolled and data from 1145 injecting events have already been collected, including 10 overdose events. This consists of 17 females and 30 males with an average age of 45 years. Data analysis is ongoing.

**Conclusions:**

This protocol establishes a foundation for advancing wearable technology in opioid overdose prevention within SIFs. The study will provide chest wall movement data and associated overdose data that will be used to train an algorithm that allows the biosensor to detect an overdose. The study will contribute crucial insights into OIRD, emphasizing the biosensor’s potential step forward in real-time intervention strategies.

**International Registered Report Identifier (IRRID):**

DERR1-10.2196/57367

## Introduction

Worldwide, more than 27 million people have an opioid use disorder and more than 150,000 people die each year from opioid overdose [1,2]. In the United States, in 2022-2023, there are estimated to be more than 100,000 drug overdose deaths [3]. Naloxone is an opioid antagonist and an antidote to opioid overdose. Effective interventions, such as take-home naloxone are available [4,5] but coverage is incomplete and they are not suited to many overdose situations, such as when people may be alone or the onset of their overdose goes unnoticed [6-8]. In such situations, intervention requires detection of the overdose to signal the need for a response.

Recently, technological developments in wireless “wearable” devices have enabled real-world, real-time measurement of physiological functioning and these devices have grown hugely in popularity [9-11]. Devices in relation to overdose could capture a wide range of different parameters but it is important that these are sensitive, accurate, and reliable. Opioids affect the control of breathing and can cause fatal overdoses, usually by respiratory failure. Respiratory depression, referring to the characteristic effect of opioid drugs, is slowed and shallow breathing (or reduced chest movement) which can sometimes lead to changes in levels of blood gasses and a suppression of the usual responses to these changes. This can lead to hypoxia, respiratory failure, and death. However, currently available wearable devices lack the ability to analyze and interpret respiratory depression data in real time and with sufficient accuracy to achieve medical device regulatory clearance. They are also not practical or economically viable for individual use in the community [12]. A chest sensor that reliably detects the overdose breathing signature, in real time, could alert family or friends to help or could directly alert emergency service. This could save lives when fully developed and used in the community.

**Figure 1:**
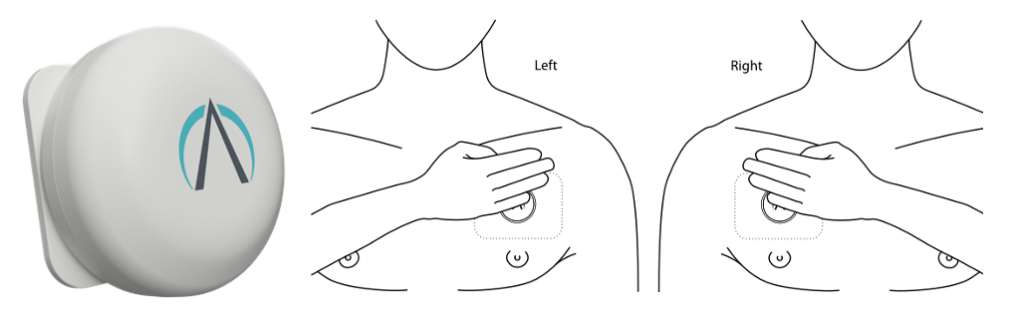
Image of PneumoWave chest sensor and location of placement on the body.

The PneumoWave chest biosensor is a small device (Figure 1; 40 mm in diameter and 14 mm in height) that sticks onto the chest using a small plastic patch (an [electrocardiographic] ECG sticker). The device measures chest motion, and consequently will be able to detect changes in chest movement. However, to be relevant to the detection of overdoses in the community, research is required on people at high risk of opioid-induced respiratory depression (OIRD) to capture chest motion during an injecting event, where the absence or presence of overdose can be externally verified. This will then be used to develop and refine sensitive and specific OIRD detection algorithms that can interpret chest movement data in real time on a smartphone or tablet without the need for live clinician analysis and interpretation.

Those who inject opioids die at a much higher rate than other drug-using groups [13]. A safe injecting facility (SIF), also known as a medically supervised injecting center or room, drug consumption room, safe consumption room, or overdose prevention center, is a legally sanctioned space where people can bring their own preobtained illegal or illicit drugs, and either inject or inhale them using sterile equipment under the supervision of nurses or other medical professionals. The main objective of a SIF is to improve survival and increase social integration [14]. However, overdoses, while at a lower rate than the general using population, still occur at SIFs [15]. This also provides an important opportunity for study [16].

In this study, as participants wear the biosensor while they are injecting their opioids, the objectives of the study are (1) to capture chest motion data using the PneumoWave biosensor, and staff-measured outcomes as the clinical reference (including clinical identification of an acute event), during instances of opioid injection and potential opioid overdose by attendees at a SIF, to develop and refine the overdose detection algorithm and (2) to examine acceptability to SIF attendees of the chest biosensor.

## Methods

### Ethical Considerations

This paper is based on version 1.1, April 5, 2023, of the study protocol. Ethics was approved by the Alfred Hospital Ethics Committee (92786). This study adheres to the Standard Protocol Items: Recommendations for Interventional Trials (SPIRIT) checklist (Multimedia Appendix 1).

In order to obtain informed consent, the site research personnel will explain the study methodology to the potential participant, the individual will be given time to read the participant information sheet and consider participation, and if they agree to participate, informed consent will be obtained. Study explanation can occur before or after using the SIF, depending on the preference of the client and aligning with the capacity to consent. Study enrollment and first recording injecting events can, therefore, occur on different days.

Data will be recorded pseudo-anonymously (ie, pseudonyms will be used; no personally identifiable information will be stored alongside chest motion data) and streamed to a secure cloud. Participants who consent to take part will be assigned a numerical study ID number that will be linked to subsequent visits that they make to the Medically Supervised Injecting Centre (MSIC) or Medically Supervised Injecting Room (MSIR). All electronic data will be anonymous and stored on secure, password-protected devices or servers, accessible only by approved study personnel. All paper-based consent forms containing participant names will be kept on site at the MSIC or MSIR following completion and will not be removed from the site.

To reimburse participants for their time and inconvenience, participants will be reimbursed Aus $50 (US $33.20) following initial screening and study enrollment. Participants will then receive a weekly reimbursement of Aus $20 (US $13.30) on the basis they have attended the MSIC or MSIR to inject opioids (either alone or in combination with other drugs) while wearing the biosensor device at least once during the week.

### Objectives

The objectives of the study are (1) to capture chest motion data using the PneumoWave biosensor, and staff-measured outcomes as the clinical reference (including clinical identification of an acute event), during instances of opioid injection and potential opioid overdose by attendees at a SIF, to develop and refine the overdose detection algorithm and (2) to examine acceptability to SIF attendees of the chest biosensor.

### Study Design

This is an observational pilot study. Over 6 months, this study aims to capture approximately 40 overdose events that occur for consenting participants who are wearing the chest sensor during visits to inject at the SIF. Participants who consent to take part will be assigned an anonymous, numerical study ID number that will be linked to subsequent visits that they make to the SIF.

Chest motion data will be collected on the device securely and pseudo-anonymously (ie, pseudonyms will be used; no personally identifiable information will be stored alongside chest motion data) and streamed to a secure cloud. A SIF is designed to respond to a broad spectrum of adverse drug reactions, recognizing that not all incidents may escalate to an opioid overdose (thus any event that is attended to and requires prompting by staff is termed an “acute event”). The data collected here will be used to test and further develop chest wall movement “signatures” to accurately detect an “acute” drug event or potential overdose, as diagnosed by the current clinical observations in the SIFs. Wearing the device will not alter the services participants receive at the SIF and the sites will manage each acute event as per their standard protocols.

### PneumoWave Biosensor and Data Collection Platform

The study will use the PneumoWave biosensor and data collection platform, which includes a wearable biosensor that attaches to the press-stud on a supplied and approved ECG electrode. The biosensor can be placed in a number of locations on the chest and diaphragm area and automatically connects to a Data Hub via Bluetooth (Figure 1). In this study, the Hub will be a router-type device or a mobile computing platform (app running on a tablet or phone).

Once consented to and enrolled in the study, participants will be asked to wear the biosensor each time they attend their relevant SIF to take opioids, an unlimited number of times. There will be no predefined number of visits that each participant should adhere to, though reimbursement will be predicated on attendance to the SIF and wearing of the biosensor at least weekly. We will now highlight the inclusion and exclusion criteria applicable to the study.

### Eligibility Criteria

#### Inclusion Criteria

Participants will be recruited from the SIFs via advertisement and direct approach by site personnel and must meet the criteria that are (1) be an adult (18 years of age or older), (2) current injecting opioid user (note: participant may be using other substances as well), (3) regular client of the SIF (minimum of 4 visits in the past month), and (4) willingness to wear biosensor device while injecting at the facility.

#### Exclusion Criteria

The exclusion criteria are (1) inability to provide informed consent, (2) younger than 18 years of age, (3) not currently injecting opioids, (4) skin sensitivity to ECG electrodes, (5) broken skin over the chest area, (6) implanted pacemaker device in situ, and (7) in the clinical opinion of the investigator would not be suitable to participate.

### Sample Size

Participants will be recruited via purposive selection—SIF staff will identify individuals who frequently attend the SIF and inject opioids—and via site advertisements, with individuals approaching site research personnel about potential participation.

During a study period of 6 months, we estimate that approximately 100 participants will be recruited at each participating SIF to meet the desired targets of approximately 2000 opioid injecting visits (a total of 4000 visits), leading to approximately 40 recorded acute or overdose events. This is based on an estimated overdose event rate (the proportion of injections that result in an overdose event) to be 1.2%, from previous data from the medically supervised injecting center in Sydney, New South Wales, Australia [15,17]). An estimated 1200 opioid injecting visits occur per month per site, with an estimated 70 unique individuals attending for opioid injecting per month, per site.

### Outcome Measures

#### Overview

We will initially separate outcome measures into categories of primary outcome measures (chest motion measures and acceptability) and SIF-determined measures. The latter will then be separated into intervention measures and acute event measures.

#### Primary Outcome Measures

Capture of chest motion of participants includes chest wall measurements via PneumoWave biosensor of people before and after injection of opioids in a SIF, compared by (1) those experiencing hypoxia and meeting defined criteria for an acute or opioid overdose (SIF-determined outcome measure or acute event) and (2) those not experiencing symptoms of hypoxia or acute or opioid overdose (ie, safe injecting event).

#### SIF-Determined Outcome Measures

The data that will be collected for each opioid injecting event in the SIF are (1) participant study ID, (2) time or date of client presentation to SIF, (3) substances reported by the participant as intended for injecting (confirmation that opioids are being injected), (4) time of opioid use, and (5) any apnea and prompts to breathe.

The outcome data that will be recorded for any intervention by SIF clinical staff (including acute event response) are (1) precise timing of the first clinical indication of acute event, (2) unique ID number of clinician initially assessing the event to be an opioid overdose, and (3) opioid overdose criteria being met (based on-site specific training materials or opioid overdose reference criteria). These are based on the measures that are described next.

The outcome measures that will be collected for each acute event (including prompts), as applicable are (1) response to audio, physical, and painful stimuli; (2) level of consciousness—timepoints as per standard of care, recorded (using Alert Verbal Pain Unresponsive [AVPU] scale—see Multimedia Appendix 2) at intervals indicated by clinical concern; (3) respiratory rate recorded at least every 5 minutes (though in practice, likely more often) after first clinical indication of opioid overdose; (4) heart rate—timepoints as per standard of care; (5) oxygen saturation via pulse oximetry were measured—timepoints as per standard of care, recorded at intervals indicated by clinical concern; (6) administration of oxygen; (7) administration of manual resuscitation or ventilate (bag-valve-mask ventilation); (8) administration of airway adjunct (nasopharyngeal or oropharyngeal airway); (9) administration of naloxone (yes or no); (10) cardiopulmonary resuscitation; and (11) ambulance called.

### Acceptability

Acceptability measures incorporate participant feedback or satisfaction survey (see Multimedia Appendix 3). During study participation, each participant will be asked at least once if they wish to provide feedback on their experience of attaching or wearing the biosensor during injecting drug use. Participants may complete the feedback questionnaire more than once to record fluctuating levels of acceptability. For any feedback provided, the site will collate responses and provide them as an anonymized table listing (see Multimedia Appendix 3). There will also be a subset of participants who will be interviewed and surveyed in more depth.

### Participant Timeline and Study Visits

The process of the investigation is more clearly understood through an examination of the schedule of events (visits 1 and then subsequent visits).

### Baseline Procedures

#### Overview

After the participant has consented, completion of participant’s basic, baseline (eg, demographics and drug use characteristics) questionnaire will be conducted using a stand-alone data collection tool using an electronic tablet. Each participant’s height and weight will be recorded at the site at baseline and changes in their height and weight will be recorded every 4 weeks.

The following schedule of events will be followed for visit 1 and then visit 2 (until withdrawal or final self-injecting event).

#### Visit 1 Procedures (Performed by Site Research Personnel)

Initially, research personnel will confirm the injection of opioids (either alone or in combination with other drugs) and then provide instructions on the use and wearing of the biosensor (Table 1). The biosensor will then be issued to the participant and the biosensor number will be recorded with the participant ID number. The researcher will verify that the biosensor has been correctly attached to the chest wall and provide further guidance to the participant, as required.

**Table 1:** Visit 1 (baseline and first study–specific self-injecting visit).

	Enrollment or baseline	Prior to injecting event	Acute or overdose event^a^	Postinjecting event	Early withdrawal^b^
Informed consent^c^	✓				
Eligibility criteria	✓				
Complete baseline questionnaire	✓				
Instructions (use of biosensor)		✓			
Issue chest sensor		✓			
Attach to chest wall		✓			
Record of event^d^			✓		
Adverse events		✓	✓	✓	✓
Return of biosensor				✓	
Early withdrawal form					✓

^a^If a self-injecting event occurred without the need for intervention the event should be recorded to reference the time of the device was attached and returned and that no interventions were required.

^b^If participant withdraws after providing consent and prior to any use of the sensor.

^c^Consent and eligibility review, performed at a time most suitable to site personnel and participant during their visit (Multimedia Appendix 4).

^d^Event start time, event stop time, assessment of event, and event outcome, if an applicable acute event occurs.

Research personnel will record basic information (day or time and drug reported for injection) for each participant’s SIF attendance. Site research personnel will record the time of self-injecting by monitoring participants from a distance and estimating the time of drug injection (this may include multiple injections within a single episode). If this is not possible to record, the site personnel will document that it could not be collected. Site research personnel will also record event interventions for an acute or overdose event (as applicable), including start time, end time, event assessment or level of intervention, clinicians’ unique ID, and overdose criteria. A record of “no acute event” will also be maintained if the self-injecting visit occurred without any requirement for site personnel intervention. Any adverse events will also be recorded and, if necessary, an early withdrawal form will be completed if the participant did not progress to use the biosensor at visit 1 and declined further participation.

#### Visit 2 to Withdrawal or Final Self-Injecting Visit (Performed by Site Research Personnel)

At visit 2 and subsequent visits until withdrawal or final self-injecting visit, research personnel will verify that the informed consent remains valid for the participant to continue on the study (Table 2). They will also confirm the injection of opioids (either alone or in combination with other drugs) and provide a reminder instruction on the use and wear of the biosensor. They will then issue the biosensor to the participant and record the biosensor number used with the participant ID. They will verify that the biosensor has been attached to the chest wall correctly and provide further guidance to the participant as required.

**Table 2:** Visit 2 to final self-injecting visit.

			Prior to injecting event		Acute or overdose^a,b^ event		Postinjecting event		Withdrawal^c^
Verify informed consent remains valid			✓						
Instructions (use of sensor; as required)			✓						
Issue chest biosensor			✓						
Attach to chest wall			✓						
Record of event^d^					✓				
Adverse events					✓		✓		✓
Return of biosensor							✓		
**User experience survey (during enrollment period, prior to withdrawal, or final self-injecting visit**									
	Withdrawal form^c^							✓	

^a^If a self-injecting event occurred without any need for intervention the event should be recorded to reference the time the device was attached and returned and that no interventions were required.

^b^If a self-injecting event occurred without the need for intervention the event should be recorded to reference the time of the device was attached and returned and that no interventions were required.

^c^For all withdrawals from visit 2 onwards.

^d^Event start time, event stop time, assessment of event, and event outcome, if an applicable acute event occurs.

Site research personnel will record basic information (day or time and drug reported for injection) for each participant’s SIF attendance and will record the time of self-injecting by monitoring the participant from a distance and estimating the time of drug injection (may include multiple injections within a single episode). If this is not possible to record, the site personnel will document that it could not be collected. Site research personnel will also record event interventions for an acute or overdose event (as applicable) including start time, end time, event assessment or level of intervention, clinicians’ unique ID, and overdose criteria. A record of “no acute event” will also be maintained if the self-injecting visit occurred without any requirement for site personnel intervention. Any adverse events will also be recorded and, if necessary, a withdrawal form will be completed if the participant did not progress to use the biosensor and declined further participation.

### Example Study Schedule

The number of visits and injection events per participant will differ. The following range of frequency of attendees at the SIF may be anticipated—frequent attendee: 2 visits per day, mid-level attendee: 5 visits per week, and low-level attendee: 1 visit per week [15,17].

### Data Management

All data will be captured using a dedicated web-based data system accessible only to site research personnel. Following completion of any given data form, data will be uploaded to a secure, password-protected server managed by the sponsor and located in Canada. It will be the Contract Research Organization’s (CRO) responsibility to manage data security. Following project completion, the CRO will lock the data system from further changes and securely transfer data to PneumoWave, where data will be securely stored. Site-specific data sets will also be provided to the SIF. All data will be anonymous, using only study IDs.

### PneumoWave Managed Biosensor Data

**Figure 2:**
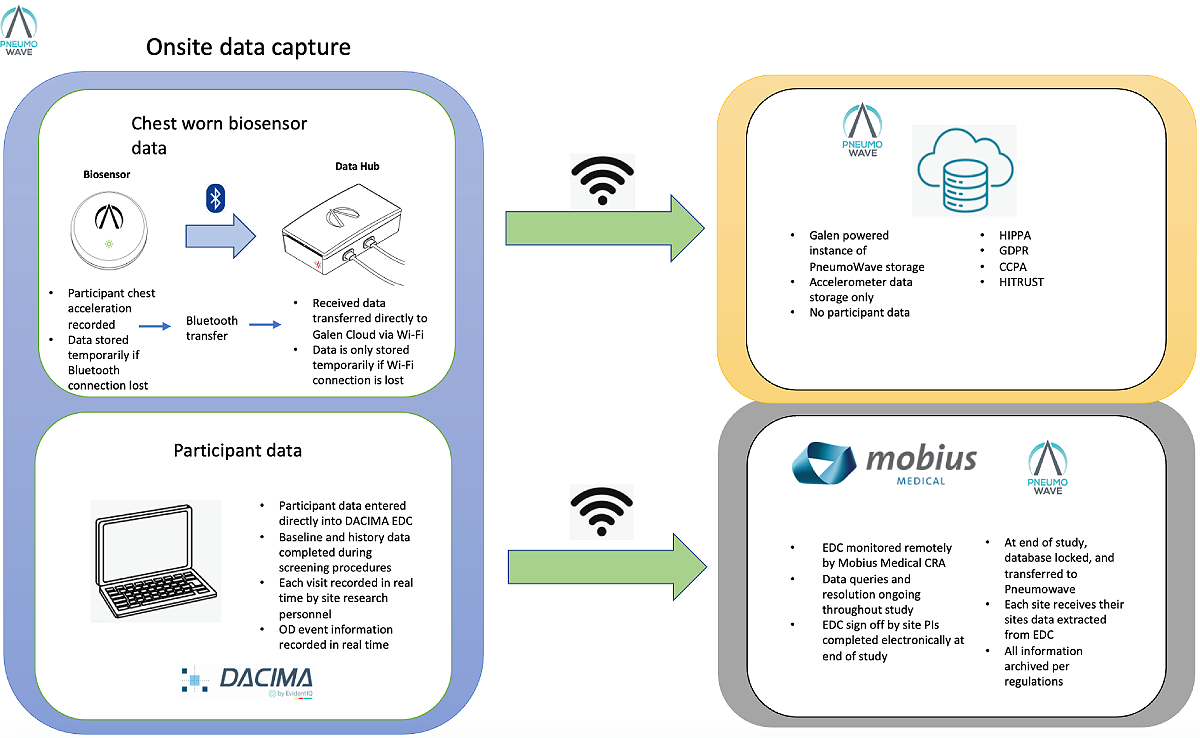
Data management flow. CCPA: California Consumer Privacy Act; CRA: Clinical Research Associate; EDC: Electronic Data Capture; GDPR: General Data Protection Regulation; HIPAA: Health Insurance Portability and Accountability Act; HITRUST: Health Information Trust Alliance; OD: overdose.

As soon as the biosensor chest placement has been carried out, chest movement data will commence being captured. Data from the biosensor will be uploaded securely and automatically to a Data Hub stored in a small container, located within the SIF (Figure 2).

The data storage platform (supplied by Galen Data) complies with HIPAA (Health Insurance Portability and Accountability Act) requirements as set forth in the Galen Cloud HIPAA Compliance Matrix (004-0020), General Data Protection Regulation (GDPR) requirements as set forth in the Galen Cloud GDPR Compliance Matrix (004-0021), and California Consumer Privacy Act (CCPA) requirements. Additionally, Galen Cloud deployed on Amazon Web Services (AWS) is Health Information Trust Alliance (HITRUST) certified.

PneumoWave will review both chest movement data stored within the Galen Cloud and study data, including baseline questionnaire, injecting event, acute event, and satisfaction questionnaire data.

### Early Withdrawal

Participants may request to withdraw from the study at any stage for any reason without prejudice. Participants will need to complete an early withdrawal form. Participants may also request deletion of all study data collected about them up until the point of their withdrawal.

### Study Procedure Risks and Benefits

It is understood there may be the occasional loss of a biosensor with the key issue being the potential for a participant to leave the facility prior to removal of the biosensor. While site personnel will aim to minimize this risk, it does remain a factor for device accountability or traceability records. The safety risk relating to a biosensor being taken off-site is assessed to be minimal. Anticipated risks are expected to be very minimal and with a low chance of occurrence. This is a noninterventional, observational study with retrospective analysis of the biosensor data and no protocol-specified procedures impacting the standard of care. Even so, potential study risks include data breaches. However, all electronic data will be anonymous and stored on secure, password-protected devices or servers, and accessible only by approved study personnel. Numerical study IDs will be used in place of participant names. All paper-based consent forms containing participant names will be kept on-site at the SIF following completion and will not be removed from the site. ECG electrodes used in the study are designed to be worn for up to 3 days continuously. The average duration of wear is expected to be less than 1 hour; however, there is a low risk of mild skin irritation.

Given the preliminary, small-scale nature of this research, it is not anticipated that there will be any direct benefits to participants. This work aims to advance the development of future devices that may identify and intervene during opioid overdose; however, such devices may not eventuate for a number of years. The absence of immediate benefit will be outlined to participants during the consent process.

### Safety

The capture of safety events will be solely related to adverse device effects (ADE) and will only be captured for an event relating to the use or wearing of the device. All ADEs and serious ADEs will be recorded by site research personnel and will be assessed for severity (mild, moderate, or severe) by the study team.

All ADEs and serious ADEs will be recorded from the time a participant is enrolled until the participant’s last study visit as per the time periods outlined. Participants who have enrolled but then withdrawn prior to wearing the biosensor for the first time will not have adverse events captured. Only unanticipated effects will be captured as reportable ADEs.

### Analyses

#### Statistical Analysis

For the SIF-determined outcome measures (eg, demographic data) and satisfaction survey, descriptive statistics will be reported using means and SD for continuous data. Absolute numbers and percentages will be reported for categorical data. Any statistical software can be used.

#### Biosensor Analyses

##### Reference Event

Staff-identified events are any event that has required an intervention by staff in the facility. Interventions can range from a simple audio stimulus, up to and including requesting an ambulance to attend. These events include those where overdose may not have occurred, but the response will have been managed in the same way according to the site’s standard operating procedures. These incidences will be recorded as an “acute event.” Where an overdose has been clearly identified using the tools mentioned previously, these incidences will be identified as “overdose event.” The analysis will incorporate both types of “reference” events.

##### Algorithm Under Test

The PneumoWave Hub receives and transmits biosensor data to a cloud storage facility. The stored data are then processed in the cloud using experimental algorithms for the presence of reduced chest movement events in adults.

The performance of the algorithm under test will be compared to the reference events at the SIF in terms of the AUT’s sensitivity and false alarm rate. If the target numbers for sample size, injecting visits, and acute events are not reached, the analysis will still be possible and feasible.

## Results

Funding was granted in July 2023 and dates for data collection are August 2023 to February 2024. As of April 2024, we have enrolled 47 participants, consisting of 17 females and 30 males with an average age of 45 years. Heroin was the preferred drug for most participants (40/47) and then methadone or an opioid agonist treatment (6/47), with the largest proportion injecting 2 to 5 times daily (BD-5D—n=23), and then once daily (14/47). The majority had experienced an overdose in the past (29/47). Among all the participants, a total of 1145 injecting events were recorded while participants wore the sensor, with 10 overdose events being observed. Data analysis of the outcome measures is ongoing.

## Discussion

There has been a recent surge of interest in mobile health and wearable devices to treat opioid use disorders [18,19] and specifically to detect and respond to opioid overdoses. However, there is limited research on detecting true overdose (which requires intervention) and the effectiveness of these devices to detect overdose in the community has not yet been adequately evaluated. A chest sensor that reliably detects the overdose breathing signature could alert the emergency services and save lives when fully developed and used in the community.

This observational study aims to capture chest motion data while participants wear a small chest-worn biosensor within medically supervised injecting centers in Australia. The data collected here will be used to test and develop respiratory “signatures” to accurately detect an acute or potential opioid overdose. Testing will allow for the differentiation of patterns that occur following opioid use but do not result in an overdose.

It is anticipated that this study will capture chest motion data from the sensor while participants inject opioids and it is likely that some instances of potential overdose will also be captured by the sensor. It is uncertain how much chest motion data will be corroborated with staff-measured outcomes. It is anticipated that the sensor will be considered positively overall among participants.

The findings of this study will be compared to a previous study of this chest sensor among participants who wear the sensor at home and are on opioid agonist treatment (paper in preparation), as well as other studies on wearable devices that have been examined acceptability and feasibility of varied technologies to combat the overdose crisis [20-25]. The potential effectiveness and acceptability of these studies are varied. Generally, there is limited but expanding research in this area with studies focused on products or technologies that are relatively new or in early development [12]. There is no published work on a remote chest sensor that can reliably detect chest wall movement in the context of an opioid overdose.

The strengths of this study are that it examines the effectiveness of the chest sensor among people who are injecting opioids in a relatively controlled but real drug-using environment. Research within an SIF is a unique opportunity to address external validity as participants will be those who the sensor is eventually intended for.

However, the study is likely to be limited in a few different ways. First, it is likely that participants will be polydrug users and it may not be possible to examine the effect of opioids alone. Additionally, the number of prompt or acute events is likely to be lower than in other drug-using environments as participants will be users who are using in a safe setting that is aimed at reducing harms and mortality around injecting drug use. This may mean the target number of acute events will be negatively impacted. Other potential limitations could include the physical movement of individuals while using the device interfering with the data capture, or data quality. The more the participants, the better the quality of data will be.

Future development of this work will involve conducting a randomized clinical investigation of a medical device to determine its effectiveness in detecting OIRD. It will also be important to test the device in people’s personal environments, for example, in their homes.

## Appendix

Multimedia Appendix 1 SPIRIT (Standard Protocol Items: Recommendations for Interventional Trials) guidelines.

Multimedia Appendix 2 AVPU (Alert Verbal Pain Unresponsive) scale.

Multimedia Appendix 3 Participant satisfaction survey.

Multimedia Appendix 4 Consent form.

## Abbreviations

ADE: adverse device effect
AVPU: Alert Verbal Pain Unresponsive
AWS: Amazon Web Services
CCPA: California Consumer Privacy Act
CRO: Contract Research Organization
ECG: electrocardiography
GDPR: General Data Protection Regulation
HIPAA: Health Insurance Portability and Accountability Act
HITRUST: Health Information Trust Alliance
MSIC: Medically Supervised Injecting Centre
MSIR: Medically Supervised Injecting Room
OIRD: opioid-induced respiratory depression
SIF: safe injecting facility
SPIRIT: Standard Protocol Items: Recommendations for Interventional Trials
