# Supplementary material for: Overdose Detection Among High-Risk Opioid Users Via a Wearable Chest Sensor in a Supervised Injecting Facility: Protocol for an Observational Study
Source: JMIR Res Protoc. 2024 Sep 10;13:e57367. doi: 10.2196/57367 (PMC11422748; doi:10.2196/57367)

Appendix I – AVPU Scale

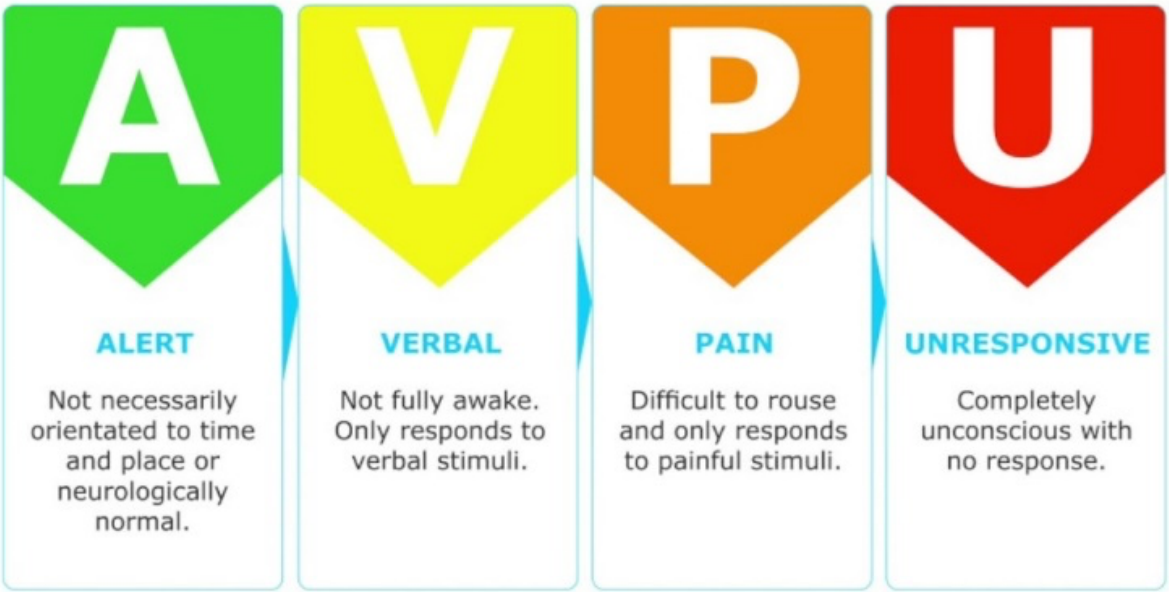

Supplement: Multimedia Appendix 2 [file resprot_v13i1e57367_app2.pdf]
