# Supplementary material for: Overdose Detection Among High-Risk Opioid Users Via a Wearable Chest Sensor in a Supervised Injecting Facility: Protocol for an Observational Study
Source: JMIR Res Protoc. 2024 Sep 10;13:e57367. doi: 10.2196/57367 (PMC11422748; doi:10.2196/57367)
Supplement: Multimedia Appendix 3 [file resprot_v13i1e57367_app3.pdf]

Appendix II Participant Satisfaction Survey

OD-SEEN

Participant Satisfaction Survey

PNEUMO WAVE

We would welcome your feedback about your experience following your wearing of the PneumoWave Biosensor today.

Based on your experience today, please answer each question by placing a circle around your score using the scale of 1 – 5.

You may also let us know more about your experience in the comment field.

	Please respond to each question by circling a number that fits best	Your comments
**Comfort**	On a scale of 1-5 how comfortable did you find this device when wearing it?<br>**1 2 3 4 5**<br>**Not at all comfortable** **Very comfortable**	
**Intrusiveness**	On a scale of 1-5 how intrusive did you find this device?<br>**1 2 3 4 5**<br>**Very intrusive** **Not at all intrusive**	
**Safety**	On a scale of 1-5 how safe did you feel wearing this device?<br>**1 2 3 4 5**<br>**Very unsafe** **Very safe**	
**Delay**	On a scale of 1-5 how much do you think the use of this device delayed your visit today?<br>**1 2 3 4 5**<br>**Significantly delayed** **No delay**	
**Normal practice**	If this device was used for your normal health care would you feel happy to wear it?<br>**1 2 3 4 5**<br>**Not at all happy** **Very happy**	
