# Supplementary material for: Overdose Detection Among High-Risk Opioid Users Via a Wearable Chest Sensor in a Supervised Injecting Facility: Protocol for an Observational Study
Source: JMIR Res Protoc. 2024 Sep 10;13:e57367. doi: 10.2196/57367 (PMC11422748; doi:10.2196/57367)
Supplement: Multimedia Appendix 4 [file resprot_v13i1e57367_app4.pdf]

CONSENT FORM

Over**D**ose **S**ensor D**E**t**E**ctio**N** among high-risk opioid users in a Medically Supervised Injection Centre (OD-SEEN)

Consent Agreement

I am over the age of 18.

I have read the Participant Information Sheet, or someone has read it to me in a language that I understand.

I understand the purposes, procedures and risks of the research described in the project.

I give permission for the staff at the injecting facility to release information to Pneumowave relating to my treatment while participating in the project I understand that such information will remain confidential.

I have had an opportunity to ask questions and I am satisfied with the answers I have received.

I freely agree to participate in this research project as described and understand that I am free to withdraw at any time during the study without affecting my future health care.

I understand that I will be given a signed copy of this document to keep.

Declaration by Participant – for participants who have read the information

Name of Participant (please print) ____

Signature_________________________________Date______________________________

Declaration by Senior Researcher
I have given a verbal explanation of the research project, its procedures and risks and I believe that the participant has understood that explanation.

Name (please print) ____

Signature__________________________________Date____________________________

Declaration of Witness – for participants unable to read the information and consent form
See Note for Guidance on Good Clinical Practice CPMP/ICH/135/95 Section 4.8.9. A legally acceptable representative may be a witness*

Witness to the informed consent process

Name (please print) ____

Signature__________________________________Date____________________________
*Witness is not to be the Investigator, a member of the study team or their delegate. Witness must be 18 years or older.

Over**D**ose Sensor D**E**t**E**ctio**N** among high-risk opioid users in a Medically Supervised Injection Centre (OD-SEEN)

If you determine you wish to withdraw your consent to participate further in this observational study please read each statement and then place your initials alongside each statement to verify you have read and agree to the statement.

WITHDRAWAL OF CONSENT

	Initials
1. I hereby wish to **WITHDRAW** my consent to participate in the study and understand that such withdrawal **WILL NOT** impact any treatment or my relationship with the injecting centre	
2. I consent to Pneumowave retaining all study data collected about me up to the date of my withdrawal.	
3. I request that all study data collected about me up to the date of my withdrawal be deleted and not used within this study.	
4. **I CONSENT* / I DO NOT CONSENT*** to continued follow up for any safety related events which have occurred during my participation in the research.	
*****_Please score through at # 4 as applicable_	

**Signature of participant**	**Please PRINT name**	**Date**
**Signature of Researcher / Investigator**	**Please PRINT name**	**Date**
**Witness (as applicable)**	**Please PRINT name**	**Date**

A copy of the Withdrawal of Consent must be Provided to the participant and the original placed on file alongside the main informed consent form.
